# Fabrication of flame retardant toughened PLA base 3D printed materials with modified lignin by phytic acid

**DOI:** 10.1039/d5ra06156c

**Published:** 2025-10-14

**Authors:** Siqi Ren, Dongfang Fan, Qian Zhang, Yunhong Jiao, Jianzhong Xu, Jixing Xie

**Affiliations:** a College of Chemistry and Materials Science, Hebei University Baoding 071002 China; b School of Eco-Environment, Hebei University Baoding 071002 China xiejx@hbu.edu.cn; c National and Local Joint Engineering Laboratory of Polymer Materials and Processing Technology Baoding 071002 China

## Abstract

Lignin, a natural macromolecule rich in aromatic rings and hydroxyl groups, holds significant promise in environmentally friendly applications. In this study, a lignin-based flame retardant (lignin–diethylenetriamine–phytic acid, P_2_N-lig) was synthesized through a Mannich reaction between alkali lignin and diethylenetriamine to introduce nitrogen, followed by phosphorylation with phytic acid to incorporate phosphorus. When P_2_N-lig were added into PLA composites (T-PLA) which were toughened by TPU and MMT, the T-PLA/P_2_N-lig composites demonstrated enhanced physicochemical and flame-retardant properties. The 9 wt% T-PLA/P_2_N-lig formulation exhibited acceptable mechanical performance alongside significantly improved thermal stability and fire resistance, achieving a 39.27% reduction in peak heat release rate (pHRR), a 22.88% decrease in total heat release (THR), and a UL-94 V-0 rating. Further evaluation of 3D-printed specimens revealed that the 6 wt% P_2_N-lig formulation achieved a limiting oxygen index (LOI) >24%, a UL-94 V-2 classification, and minimal mechanical degradation, demonstrating suitability for 3D printing applications.

## Abstract 2

A flame-retardant and toughened PLA-based material suitable for 3D printing was prepared by modifying lignin with phytic acid and diethyltriamine and combining it with toughened PLA.

## Graphical abstract

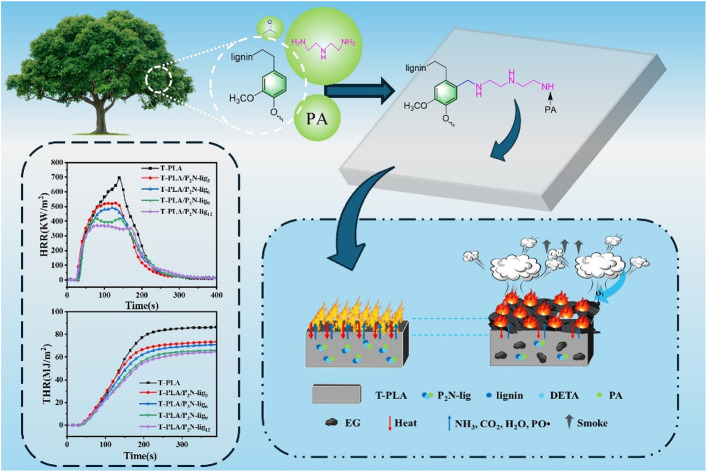

## 1. Introduction

With the environmental impact of traditional petroleum-based plastics, there is an imperative need to develop bio-based biodegradable materials^1^ as alternatives to conventional plastics.^2^ Polylactic acid (PLA), a bio-based plastic^3^ polymerized from numerous lactic acid units with ester bonds,^4^ is highly valued for its biodegradability,^5^ renewable raw materials,^6^ low-carbon emissions,^7^ and good biocompatibility,^8^ and is widely used in 3D printing^9^ and medical devices.^10^ However, PLA's mechanical properties are subpar,^11^ and it has almost no flame retardant properties,^12^ necessitating modification with other materials for diverse applications.^13,14^

Lignin, a bio-based macromolecule,^15^ is highly regarded in the field of flame-retardancy.^16,17^ It is rich in aromatic ring structures and has high carbon content.^18^ These characteristics enable lignin to form a stable char layer during combustion,^19^ effectively insulating heat and oxygen, thus inhibiting combustion.^20^ The high-temperature decomposition and strong thermal stability of lignin further delay the thermal decomposition process of materials.^21^ After modification, lignin exhibits good compatibility with various polymers (such as PLA, PP, PE) ^22–24^ and has a certain melt-flow property at high temperatures, making it suitable for the preparation of composites *via* melt-blending.^25^ Lignin suppresses combustion through a condensed-phase flame-retardant mechanism and free-radical trapping.^26^ As a renewable resource, lignin is environmental friendly and low-in-toxicity.^27^ Moreover, lignin can synergize with other flame retardants,^28–30^ and its compatibility with polymers can be enhanced through modification,^31^ making it suitable for melt-processing.^32^ These attributes render lignin a highly efficient, multifunctional, and sustainable flame^33^ retardant with great potential for application in the field of fire-retardancy.^34^

Currently, main lignin modification methods include Mannich reaction, sulfonation modification, and physical blending.^35^ Zhang *et al.*^36^ employed tetraethylenepentamine and DOPO to modify homemade lignin oligomers, introducing P and N elements into lignin. Subsequent blending with PLA yielded composites that achieved a V0 rating at 10% flame-retardant loading. Zhang *et al.*^37^ blended APP with lignin and incorporated the mixture into PLA. The composite, containing 23% flame retardant, only reached a V2 rating due to molten droplets. However, when APP was blended with urea-modified lignin and added to PLA, the composite attained V0 performance. Meng *et al.*^38^ fabricated composite PLA films by incorporating sodium lignosulfonate-modified carbon nanotubes into PLA, achieving a 194.5% increase in the elongation at break.

In this study, a novel green and efficient lignin-based flame retardant (P_2_N-lig, lignin–diethylenetriamine–phytic acid) was synthesized. Lignin was reacted with diethylenetriamine *via* a Mannich reaction to graft N-containing groups firstly, followed by the incorporation of phytic acid to introduce P-containing groups. This novel flame retardant was then added to a toughened PLA to create a flame-retardant 3D printing material. Tests on the flame retardant and the 3D-printed material showed improved flame retardancy.

## 2. Experimental

### 2.1 Materials and reagents

Alkaline lignin (AL) was purchased from Aladdin Bio-Tech Co., Ltd (Shanghai, China). Diethylenetriamine (C_4_H_13_N_3_) and phytic acid (70%) were obtained from Macklin Bio-Tech Co., Ltd (Shanghai, China). PLA was supplied by Anhui Fuyuan Futai Lai Polylactic Acid Co., Ltd. Chemicals such as formaldehyde, sulfuric acid (H_2_SO_4_), and sodium hydroxide (NaOH) were purchased from Tianjin Damao Chemical Reagent Co., Ltd, China, and used without further purification.

### 2.2 Synthesis of lignin-diethylenetriamine (N-lig)

Dissolve AL (5.0 g) in 10.0 mL of 0.4 mol per L NaOH, add diethylenetriamine (39.0 g, 0.38 mol) and formaldehyde (30.0 mL, 37 wt%), and react in a 60 °C water bath for 3 hours. Cool the solution to 0 °C, add H_2_SO_4_ dropwise, centrifuge the precipitated N-lig, and dry it overnight at 40 °C to obtain a brown powder.

### 2.3 Synthesis of lignin–diethylenetriamine–phytic acid (P_2_N-lig)

The mixture of lignin-N and phytic acid at a molar ratio of 2 : 1 was reacted for 2 hours, followed by rotary evaporation. The resultant sample was subsequently washed with anhydrous ethanol and subjected to centrifugation. The purified product was then dried in an oven at 60 °C to obtain P_2_N-lig.

### 2.4 Preparation of toughened PLA/P_2_N-lig blends (T-PLA/P_2_N-lig)

All samples were dried at 70 °C for 4 hours. T-PLA/P_2_N-lig composites with P_2_N-lig loadings of 0, 3, 6, 9, and 12 wt% were fabricated *via* a twin-screw extruder (CTE20, Coperion (Nanjing) Machinery Co., Ltd). The temperature of the extruder barrel zones (Z1 to Z6) were maintained at 160 °C, 170 °C, 175 °C, 170 °C, 170 °C, and 165 °C, respectively. The extrudates were pelletized and subjected to subsequent characterization.

### 2.5 Preparation of T-PLA/P_2_N-lig 3D printing materials

Toughened polylactic acid (T-PLA) and flame retardant were initially dried at 70 °C for 4 hours to eliminate moisture, thereby preventing bubble formation or thermal degradation during processing. The components were melt-blended at 170 °C using a twin-screw extruder with P_2_N-lig loadings of 0, 3, 6, 9, and 12 wt%, yielding homogeneous 3D printing filaments. Subsequently, fused deposition modeling (FDM) 3D printing was performed at a nozzle temperature of 190–210 °C and a heated bed temperature of 50–60 °C. Post-printing, the specimens were annealed at 80–100 °C for 2–4 hours to relieve internal stresses, followed by optional surface treatment as required for specific applications. For the tensile tests, specimens with an effective gauge section dimensions of 25 mm × 4 mm × 2 mm were fabricated with 16 printing layers, 100% infill density, a base layer printing speed of 50 mm s^−1^, and a travel speed of 70 mm s^−1^. Impact and oxygen index tests employed specimens measuring 80 mm × 10 mm × 4 mm, printed with 25 layers, 100% infill density, a base layer speed of 60 mm s^−1^, and a travel speed of 80 mm s^−1^. UL94 vertical burning tests were conducted on specimens sized 125 mm × 13 mm × 2.7 mm, which were printed with 16 layers, 100% infill density, a base layer speed of 60 mm s^−1^, and a travel speed of 80 mm s^−1^.

### 2.6 Characterization

#### 2.6.1 Fourier transform infrared spectroscopy

FTIR spectroscopy analysis was conducted using a Tensor 27 Fourier transform infrared spectrometer (Bruker, Germany) with a spectral range of 4000–400 cm^−1^ and a resolution of 2 cm^−1^. Samples were prepared *via* the KBr pellet method to characterize the variations in characteristic functional groups of the materials.

#### 2.6.2 Scanning electron microscopy (SEM) characterization

The surface morphology of the samples was examined using a field-emission scanning electron microscope (FE-SEM, TM-3000) operated at an acceleration voltage of 5.0 kV. Prior to imaging, the samples were sputter-coated with a gold layer (15 mA, 30 s) to enhance conductivity. Observations were conducted under a working distance of 8–10 mm with magnification levels ranging from one thousand to two thousand times.

#### 2.6.3 Thermogravimetric analysis (TGA)

Thermogravimetric analysis (TGA) was performed on a STA449C thermal analyzer under a N_2_ atmosphere (flow rate: 50 mL min^−1^) with a temperature ramp from 30 °C to 800 °C at a heating rate of 10 °C min^−1^. The mass loss profiles of the samples were recorded as a function of temperature.

#### 2.6.4 Limiting oxygen index (LOI) test

The limiting oxygen index (LOI) of the materials was determined in accordance with the GB/T 2406.2-2009 standard using an oxygen index tester (Phoenix Quality Testing Instrument Co., Ltd). Specimens with dimensions of 100 mm × 6.5 mm × 3.0 mm were tested under controlled environmental conditions at 23 ± 2 °C and 50 ± 5% relative humidity.

#### 2.6.5 Mechanical properties testing

Tensile properties were evaluated in accordance with the international standard ISO 527 using a universal testing machine (CMT2503) at a crosshead speed of 1 mm min^−1^. Dumbbell-shaped specimens with an effective gauge section of 25 mm × 4 mm × 2 mm and rectangular impact testing bars measuring 80 mm × 10 mm × 4 mm were tested, and a minimum of five replicates per group were measured to ensure statistical reliability.

#### 2.6.6 Cone calorimeter test

Fire behavior was evaluated using a cone calorimeter (iCone Plus, Fire Testing Technology Ltd) following the ISO 5660-1 standard. Specimens with dimensions of 100 mm × 100 mm × 3 mm were exposed to a radiant heat flux of 35 kW m^−2^, and key fire reaction parameters including heat release rate (HRR), total heat release (THR), and smoke production rate (SPR) were systematically recorded.

#### 2.6.7 UL94 test

Vertical burning tests were conducted according to the UL 94 standard. Specimens with standardized dimensions (125 mm × 13 mm × 3 mm) were vertically clamped in a holder, positioned 300 mm above a layer of degreased cotton. The bottom edge of the specimen was ignited with a 20 mm-high flame for 10 seconds. The afterflame time was recorded until self-extinction. If self-extinguishing occurred, the specimen was reignited for an additional 10 seconds, and the total afterflame time was measured. Simultaneously, the ignition capability of molten drips on the cotton was monitored. The material's flammability rating (V-0, V-1, or V-2) was assessed based on the cumulative after flame duration and dripping behavior.

#### 2.6.8 Raman spectroscopy

The sample was placed on a microscope stage, and a suitable area was selected. A laser with a specific wavelength (*e.g.*, 532 nm or 785 nm) was used to irradiate the sample, and the scattered light was collected. The frequency shifts of the scattered light were analyzed *via* a spectrometer to obtain the Raman spectrum.

#### 2.6.9 ^31^P NMR spectra analysis

Nuclear magnetic resonance spectra were performed on a Bruker AVANCEIII NMR spectrometer (600 MHz) at room temperature. The solvent used was deuterated dimethyl sulfoxide (DMSO-*d*_6_).

## 3. Results and discussion

### 3.1 Analysis of P_2_N-lig

**Fig. 1:**
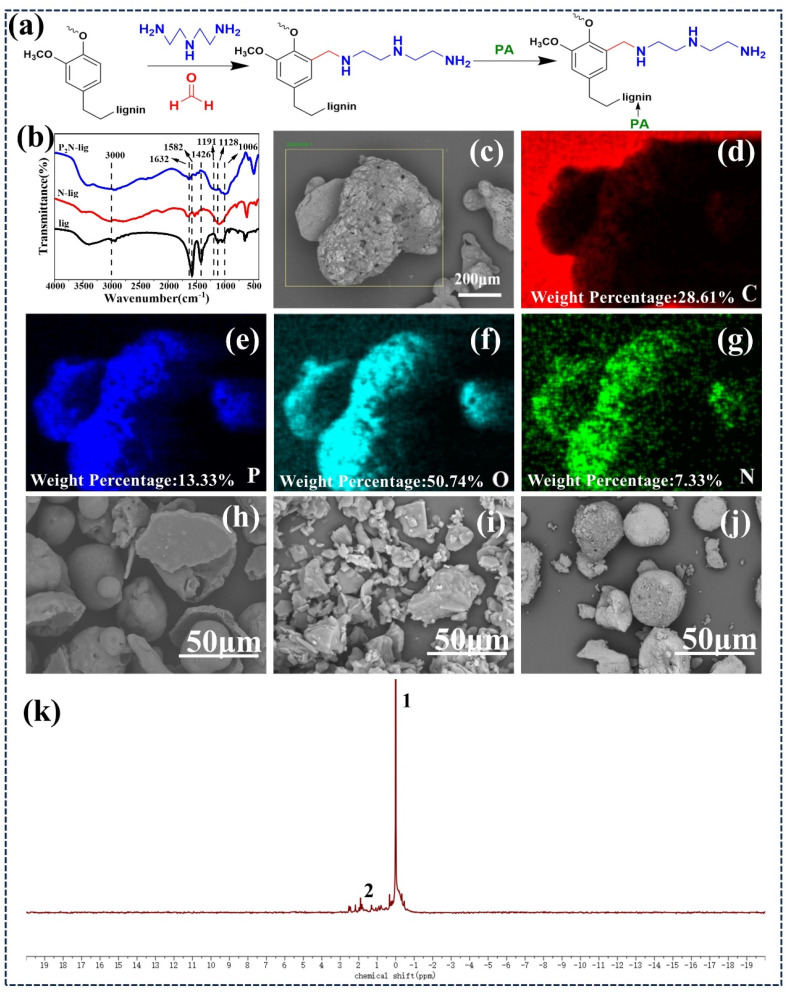
(a) Synthetic route of P_2_N-lig; (b) FT-IR spectra; (c–g) chemical element mapping of P_2_N-lig; SEM images of (h) lignin, (i) N-lig, (j) P_2_N-lig, (k) ^31^P NMR spectra.

In this study, a Mannich reaction was employed to graft diethylenetriamine onto lignin, followed by the incorporation of phytic acid to introduce phosphorus species, as illustrated in the synthetic strategy shown in Fig. 1a. Fig. 1b presents the FTIR spectra of lignin (lig), amine-modified lignin (N-lig), and phosphorylated lignin (P_2_N-lig). The absorption bands near 3000 cm^−1^ are attributed to C–H stretching vibrations of methyl or methylene groups,^39^ while the peaks between 1426 cm^−1^ and 1582 cm^−1^ correspond to aromatic C–H bending vibrations. The characteristic peak at 1128 cm^−1^ arises from ether bond vibrations in lignin, reflecting its primary structure. For N-lig, the emergence of a peak at 1632 cm^−1^ indicates N–H bending vibrations of amine groups. Additionally, minor shifts and intensity reduction in aromatic C–H vibrations (1426–1582 cm^−1^) compared to original lignin confirm the successful grafting of diethylenetriamine at the C3 and C5 positions of phenolic units. In the P_2_N-lig spectrum, new peaks at 1191 cm^−1^ and 1006 cm^−1^ are assigned to P=O stretching vibrations and P–O–C absorption bands respectively, demonstrating the covalent binding of phytic acid to N-lig.^40^ The ^31^P NMR spectrum (Fig. 1k) revealed a strong signal at Position 1 (approximately 0 ppm), indicative of the presence of P(=O)–O bonds.^41^ Additionally, multiple peaks were observed at Position 2 (around 2.5 ppm), which can be attributed to the restricted rotation of P–O–C bonds due to the rigid structure of the lignin macromolecule. This rigidity results in heterogeneous local electronic environments, causing slight downfield shifts for different P–O–C sites owing to variations in shielding effects. The coexistence of P(=O)–O and P–O–C bonds further confirms the successful covalent grafting of phytic acid onto N-lig. As confirmed by the elemental mapping in Fig. 1c–f and Table S1, the successful incorporation of N (7.33 wt%) and P (13.33 wt%) has been achieved. The combined spectral data provide confirmation of the successful synthesis of P_2_N-lig.

SEM characterization was performed on lig, N-lig, and P_2_N-lig, as shown in Fig. 1g–j. Fig. 1g reveals that pristine lignin (lig) has a spherical shell-like structure. Upon diethylenetriamine modification (N-lig, Fig. 1i), the microstructure transitions into irregular blocks with significantly reduced dimensions. Further phytic acid functionalization (P_2_N-lig, Fig. 1j) induces micro-void formation on the surface, accompanied by further size reduction and more homogeneous particle distribution.

**Fig. 2:**
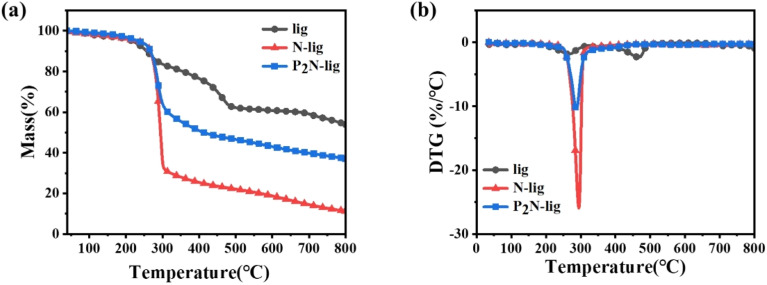
Thermogravimetric curves of P_2_N-lig under N_2_ atmosphere: (a) TGA, (b) DTG.

Through the comparative investigation of the thermal degradation behaviors of lig, N-Lig, and P_2_N-lig (Fig. 2 and Table S2), this study elucidates the regulatory mechanism of nitrogen-phosphorus synergistic modification on lignin thermal stability. Thermogravimetric analysis under N_2_ atmosphere revealed that functional modification significantly enhanced the *T*_5%_ (temperature at 5% mass loss) of lignin. The *T*_5%_ of unmodified lig was 216.3 °C, whereas those of N-lig and P_2_N-lig increased to 228.3 °C and 232.7 °C respectively, representing 5.6% and 7.6% improvement over the pristine sample, confirming the constructive effect of modification in establishing thermally stable functional groups.

Notably, significant shifts in *T*_max_ (temperature at maximum decomposition rate) were observed. While the unmodified lig exhibited a *T*_max_ of 466.7 °C, those of N-lig and P_2_N-lig decreased to 294.5 °C and 287.8 °C respectively. This phenomenon can be attributed to the preferential thermal cleavage of nitrogen/phosphorus-containing functional groups, which facilitates early release of non-combustible gases (*e.g.*, NH_3_, H_2_O) and promotes char layer formation, thereby achieving flame-retardant synergism.

The evolution of char residue further corroborates the modification mechanism. The unmodified lig exhibited a char yield of 53.8% at 800.0 °C, whereas that of N-lig dramatically decreased to 11.6%, suggesting that the nitrogen component's free radical quenching effect accelerates polymer chain scission. In contrast, P_2_N-lig demonstrated a significantly enhanced char yield of 37.1%, representing a 219.8% increase relative to N-lig. This improvement is attributed to phosphorus-catalyzed dehydration reactions and the promotion of cross-linked network formation, which facilitates the development of a compact char layer that effectively obstructs heat and mass transfer.

### 3.2 Thermal stability analysis of T-PLA/P_2_N-lig FR composites

**Fig. 3:**
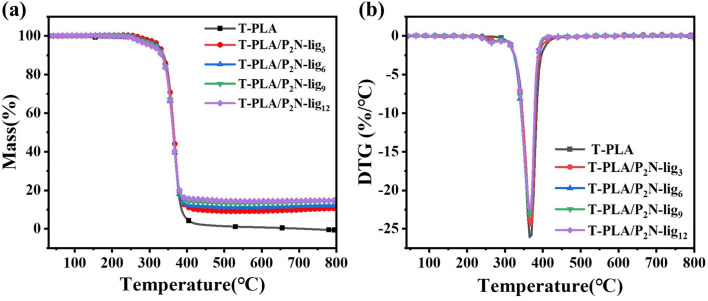
Thermogravimetric curves of T-PLA/P_2_N-lig under N_2_ atmosphere: (a) TGA, (b) DTG.

To further evaluate the thermal stability of P_2_N-lig in T-PLA composites, thermogravimetric analysis was conducted, as illustrated in Fig. 3 and Table S3. P_2_N-lig was incorporated into T-PLA at varying loadings (3 wt%, 6 wt%, 9 wt%, and 12 wt%) to investigate the optimal formulation. As shown in Table S3, the *T*_5%_ of pristine T-PLA was 324.0 °C. When a small amount of P_2_N-lig (*e.g.*, 3 wt%, denoted as T-PLA/P_2_N-lig_3_) was introduced, the *T*_5%_ slightly increased to 324.4 °C, indicating minimal impact on the initial decomposition temperature of the matrix. However, the *T*_5%_ gradually decreased with higher P_2_N-lig loading.

This downward trend might be attributed to two factors: (1) the incorporation of P_2_N-lig could disrupt the structural regularity of T-PLA molecular chains, thereby compromising thermal stability, and (2) P_2_N-lig itself undergoes decomposition at lower temperatures compared to T-PLA (as evidenced by the TGA data of pure P_2_N-lig in Table S3). This premature decomposition of the additive initiates significant thermal decomposition of the composite at reduced temperatures, resulting in diminished initial thermal stability of the material.

The *T*_max_ of pristine T-PLA was 363.4 °C. Upon incorporation of P_2_N-lig, minor fluctuations in *T*_max_ values were observed across composite formulations. However, all modified systems maintained *T*_max_ values consistently higher than that of unmodified T-PLA. These results conclusively demonstrate that the addition of P_2_N-lig stabilizes the thermal stability of the composite material during its principal decomposition phase, effectively preventing premature drastic decomposition.

Regarding residual mass analysis, pristine T-PLA exhibited an extremely low char yield of 0.5% under N_2_ atmosphere, which arises from its thermal decomposition mechanism dominated by random chain scission of molecular structures, generating volatile small molecules with minimal residue. In contrast, the incorporation of P_2_N-lig significantly enhanced the residual char formation. The T-PLA/P_2_N-lig_3_ composite demonstrated a char yield of 10.65%, while the T-PLA/P_2_N-lig_12_ system achieved a notably higher value of 14.73%. Furthermore, the char yield exhibited a progressive upward trend with increasing P_2_N-lig loading.

This phenomenon indicates that P_2_N-lig substantially improves the thermal stability of the composite material. The enhancement mechanism can be attributed to phosphorus-derived species generated during high-temperature decomposition (*e.g.*, phosphoric acid or polyphosphoric acid), which catalyze the dehydration and carbonization of lignin. These acidic compounds promote the formation of a compact char layer endowed with exceptional thermal insulation and oxygen barrier properties. The resultant char layer effectively impedes heat transfer and oxygen diffusion, thereby suppressing further thermal degradation and combustion of the material.

### 3.3 Flame-retardancy analysis of T-PLA/P_2_N-lig FR composites

As shown in the limiting oxygen index (LOI) data on the Table 1, the T-PLA exhibited a low LOI value of 20.5%, indicative of poor flame retardancy. Upon incorporation of P_2_N-lig, the LOI values of the composites showed marked enhancement, with a progressive increase paralleling the rising P_2_N-lig loading. Notably, T-PLA/P_2_N-lig_9_ and T-PLA/P_2_N-lig_12_ achieved LOI values exceeding 25%, reaching the threshold for self-extinguishing materials. This trend unequivocally demonstrates that the introduction of P_2_N-lig substantially enhances the flame retardancy of T-PLA-based composites, requiring higher oxygen concentrations to sustain combustion.

**Table 1:** The LOI and UL94 test data of T-PLA/P_2_N-lig

Samples	LOI (%)	*t*_1_ (s)	*t*_2_ (s)	Whether there are molten droplets	Rating
T-PLA	20.5	—	—	Yes, the absorbent cotton is ignited	NO
T-PLA/P_2_N-lig_3_	22.1	12.1	5.8	Yes, the absorbent cotton is ignited	V-2
T-PLA/P_2_N-lig_6_	23.0	11.1	4.3	Yes, the absorbent cotton is ignited	V-2
T-PLA/P_2_N-lig_9_	25.0	10.1	1.5	Yes, but the absorbent cotton is not ignited	V-0
T-PLA/P_2_N-lig_12_	26.5	8.2	1.1	NO	V-0

As demonstrated by the UL-94 vertical burning test results on Table 1, pristine T-PLA failed to pass the evaluation. However, the combustion behavior of the composites improved significantly upon the incorporation of P_2_N-lig. With increasing P_2_N-lig loading, the T-PLA/P_2_N-lig_12_ composite exhibited a reduced first after flame time (*t*_1_) to 8 seconds and a second after flame time (*t*_2_) to 1.1 seconds, accompanied by the elimination of melt dripping. This trend clearly indicates that the addition of P_2_N-lig effectively shortens the sustained combustion duration and remarkably enhances the self-extinguishing properties of the material.

The addition of 3 wt% P_2_N-lig enabled the composite to achieve a V-2 rating, while a higher loading of 9 wt% P_2_N-lig elevated the rating to V-0. These outcomes unequivocally confirm that P_2_N-lig incorporation significantly enhances the flame retardancy of T-PLA-based composites, transforming them from poorly flame-retardant materials to systems with superior fire resistance. This improvement is reflected not only in reduced combustion times and suppressed melt dripping but also in the substantial elevation of fire safety ratings, providing robust support for the application of T-PLA in flame-retardant fields.

### 3.4 Combustion performance analysis of T-PLA/P_2_N-lig FR composites

**Fig. 4:**
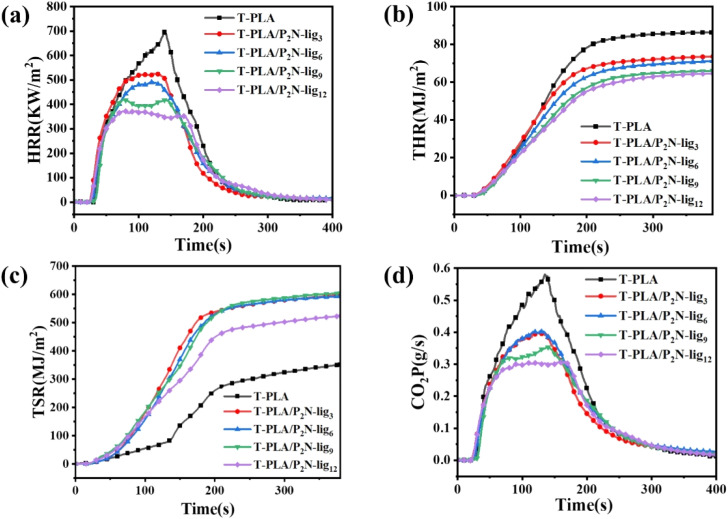
Cone calorimetry profiles of T-PLA/P_2_N-lig: (a) HRR, (b) THR, (c) TSR, (d) CO_2_P.

Cone calorimetry data (Fig. 4 and Table S4) revealed that minimal P_2_N-lig loading (3 wt%) slightly reduced the material's ignition time (TTI), while higher loadings stabilized or even prolonged TTI. This phenomenon is attributed to the dense char layer formed through catalytic carbonization by P_2_N-lig, which partially impeded oxygen and heat transfer during initial combustion stages. Such barrier effects counterbalanced the accelerated gas-phase reactions, ultimately stabilizing ignition characteristics. The TTI modulation demonstrates the progressive dominance of condensed-phase flame retardancy mechanisms over gaseous-phase interactions as P_2_N-lig concentration increases.

The peak heat release rate (pHRR) of T-PLA is 691.04 kW m^−2^. As the amount of P_2_N-lig added increases, the pHRR shows a significant downward trend. When 12 wt% P_2_N-lig is added, the pHRR of the composite material decreases by 46.19%. This indicates that P_2_N-lig can effectively reduce the intensity of the composite material during combustion, thus decreasing thermal hazards in fires. The total heat release (THR) of T-PLA is 86.6 MJ m^−2^. After adding P_2_N-lig, the THR of T-PLA/P_2_N-lig_12_ drops to 65.08 MJ m^−2^, showing that P_2_N-lig reduces the total heat released during material combustion, thereby lessening the overall fire hazard. This may be attributed to the phosphorus components derived from phytic acid in P_2_N-lig, which promote the crosslinking and carbonization of T-PLA molecular chains, while the lignin framework provides skeletal support for the carbon layer. Together, they form a continuous and dense protective layer, inhibiting the combustion of the composite material.

The residual mass of T-PLA is only 1.23%. After adding P_2_N-lig, the residual mass increases significantly. When at least 9 wt% flame retardant is added, it significantly promotes char layer formation. The aromatic structure and phosphorus–nitrogen synergistic catalytic effect of P_2_N-lig enhance the thermal stability of the char layer. In terms of gas release, P_2_N-lig significantly reduces the generation rate of CO_2_ (CO_2_P) but has a smaller impact on the generation rate of CO (COP), indicating a tendency to inhibit the formation of complete combustion products. P_2_N-lig improves the flame retardancy of T-PLA through synergistic gas-phase and condensed-phase actions.

### 3.5 Char residue analysis of T-PLA/P_2_N-lig FR composites

**Fig. 5:**
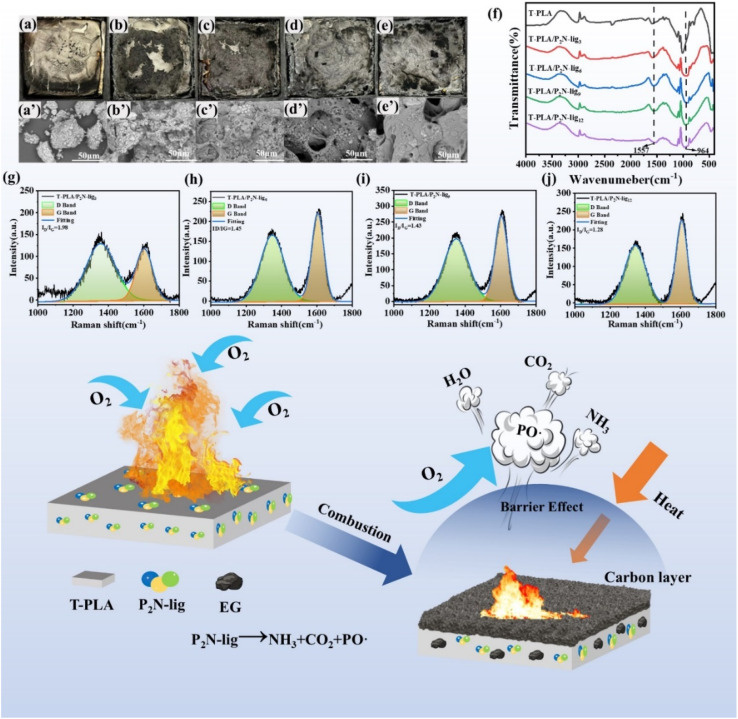
Char residue analysis of T-PLA/P_2_N-lig FR composites.

Macroscopic characterization of char residues (Fig. 5a–e) revealed distinct morphological differences. Neat T-PLA exhibited smooth carbonaceous surfaces with visible cracks and fragmentation (Fig. 5a), indicative of structural instability during combustion that prevented the formation of continuous, robust char layers – a key factor in its poor flame retardancy. In contrast, P_2_N-lig-modified composites demonstrated progressively rougher surfaces with enhanced porosity and irregular cavities as additive loading increased (Fig. 5b–e). This morphological evolution suggests that P_2_N-lig modulates the carbonization process, facilitating the development of interconnected porous carbonaceous structures.

SEM analysis (Fig. 5a′–e′) revealed significant microstructural evolution of char residues. Pristine T-PLA exhibited loosely packed carbonaceous particles with weak interparticle connectivity and minimal agglomeration (Fig. 5a′), a structural configuration that fails to impede heat/oxygen transfer, thereby limiting flame-retardant efficacy. Conversely, P_2_N-lig-incorporated composites demonstrated progressive densification and structural ordering (Fig. 5b′–e′). At elevated additive loadings, the char residues developed interconnected architectures with continuous network-like porosity, suggesting enhanced crosslinking during thermal degradation. This optimized microstructure facilitates the formation of a coherent thermal-oxidative barrier, effectively retarding heat propagation and suppressing volatile.

FTIR analysis of char residues (Fig. 5f) revealed structural differences in carbonized phases. The pristine T-PLA exhibited weak absorption bands in the characteristic aromatic C–C stretching vibration region, suggesting limited development of thermally stable graphitic structures. With increasing P_2_N-lig content, significant enhancement of these absorption features was observed, indicative of promoted aromatic crosslinking during thermal degradation. This structural evolution facilitates the formation of condensed char layers with improved thermal stability, which effectively restrict heat transfer and oxygen diffusion through physical barrier effects during combustion. The absorption band corresponding to C–O stretching vibrations (observed at 964 cm^−1^ in pristine T-PLA) exhibited notable intensity variations and potential band shifting with increasing P_2_N-lig content. These spectral modifications suggest that P_2_N-lig influences the formation, cleavage, and distribution of C–O bonds during combustion. The evolving peak characteristics likely originate from specific interactions between functional groups in P_2_N-lig (*e.g.*, phosphorus/nitrogen moieties) and oxygenated carbon structures in the char matrix, ultimately governing the structural integrity and flame-retardant functionality of the carbonaceous residues.

Raman spectroscopy analysis of char residues (Fig. 5g–j) demonstrated structural optimization induced by P_2_N-lig. With increasing additive content, the intensified G-band intensity and reduced D/G ratio revealed progressive graphitization of the carbonaceous residues. This structural evolution indicates enhanced ordering of graphitic domains and decreased structural defects within the char matrix. The improved structural integrity of the modified residues correlates with superior thermal stability and oxygen/heat barrier capability. Such ordered carbon architectures effectively retard heat conduction and gas diffusion during combustion, thereby amplifying the flame-retardant performance through optimized condensed-phase shielding mechanisms.

The P_2_N-lig flame retardant, synthesized from lignin, diethylenetriamine, and phytic acid, possesses a molecular structure rich in nitrogen, phosphorus, and aromatic components, endowing it with dual-phase flame-retardant capabilities (Fig. 5). In the gas-phase mechanism, P_2_N-lig decomposes during combustion to release nitrogen- and phosphorus-containing gases (*e.g.*, NH_3_, PO· radicals), which dilute flammable volatiles (*e.g.*, lactic acid, acetaldehyde) generated by T-PLA degradation and interrupt free-radical chain reactions. Simultaneously, in the condensed-phase mechanism, P_2_N-lig promotes the formation of a dense and continuous char layer at high temperatures, effectively isolating the polymer matrix from oxygen and heat transfer while suppressing the release of combustible volatiles. These two mechanisms synergistically enhance the flame retardancy of T-PLA: gas-phase inhibition dominates during the initial combustion stage by suppressing flammable gas ignition, while the condensed-phase barrier effect becomes predominant in later stages by physically blocking heat and oxygen diffusion. Experimental results confirm the effectiveness of this dual mechanism, as increasing P_2_N-lig content significantly improves char yield and reduces combustion rate in T-PLA composites.

### 3.6 Mechanical property analysis of T-PLA/P_2_N-lig FR composites

**Fig. 6:**
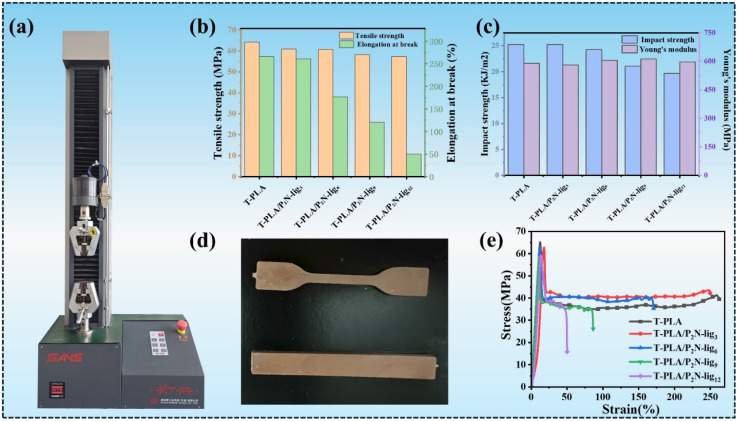
Mechanical properties of T-PLA/P_2_N-lig: (a) optical images of the CMT2503 equipment, (b) tensile testing, (c) impact testing and Young's modulus, (d) test spline optical photographs, (d) stress–strain curves.

Fig. 6 presents the comparative mechanical analysis of T-PLA and its composites with varying P_2_N-lig loadings, including tensile strength, elongation at break, impact strength and their stress–strain curves.

The pristine T-PLA exhibits a tensile strength of approximately 64 MPa, demonstrating robust mechanical performance. As the P_2_N-lig content increases from T-PLA/P_2_N-lig_3_ to T-PLA/P_2_N-lig_12_, the tensile strength progressively decreases, reaching approximately 57 MPa for T-PLA/P_2_N-lig_12_. This reduction is primarily attributed to the limited interfacial compatibility between P_2_N-lig and the T-PLA matrix. At lower loadings, the additive minimally disrupts the ordered arrangement of T-PLA molecular chains, resulting in negligible changes in tensile strength. However, higher P_2_N-lig concentrations lead to inhomogeneous dispersion within the matrix, creating localized stress concentration points. These defects impede the coordinated deformation of polymer chains during tensile loading, ultimately diminishing the composite's tensile strength.

The pristine T-PLA exhibits an elongation at break of approximately 280%, indicating favorable flexibility. However, increasing P_2_N-lig content significantly reduces this parameter, with T-PLA/P_2_N-lig_3_ and T-PLA/P_2_N-lig_12_ showing respective decreases to ∼260% and ∼60%. This trend arises from restricted molecular chain mobility induced by P2N-lig incorporation. Potential hydrogen bonding or physical entanglement between P_2_N-lig and T-PLA chains reduces segmental freedom, limiting large-scale deformation under stress. Furthermore, the inherent rigidity of P_2_N-lig particles compromises the matrix's deformability, creating heterogeneous regions that preferentially initiate crack propagation during elongation.

Regarding impact strength, pristine T-PLA demonstrates a value of ∼25.5 kJ m^−2^, which progressively decreases with higher P_2_N-lig loadings, reaching ∼19.5 kJ m^−2^ for T-PLA/P_2_N-lig_12_. This decline stems from interfacial effects introduced by P_2_N-lig. The incorporation of P_2_N-lig increases internal interfaces within the composite, which act as preferential sites for crack initiation and propagation under impact loading. At lower additive concentrations, interfacial effects remain negligible, resulting in minimal changes to impact strength. However, higher P_2_N-lig loadings amplify interfacial defects, facilitating crack propagation and reducing energy dissipation capacity. Furthermore, insufficient interfacial adhesion between P_2_N-lig particles and the T-PLA matrix may induce interfacial debonding during impact, exacerbating the deterioration of toughness.

The Young's modulus at varying addition ratios of P_2_N-lig is presented in Fig. 6c. The neat T-PLA exhibits a Young's modulus of approximately 589.7 MPa. With the incorporation of P_2_N-lig, the Young's modulus of the composite material demonstrates a generally increasing trend. At a 3 wt% loading, P_2_N-lig temporarily functions as a plasticizer, weakening the intermolecular interactions within the PLA matrix and resulting in a slight reduction in stiffness. As the additive content progressively increases, P_2_N-lig acts as a rigid filler dispersed in the T-PLA matrix, thereby enhancing its stiffness. However, further increase in its concentration promotes the formation of stress concentration points, leading to a deterioration in toughness. At a 12 wt% loading, the agglomeration of P_2_N-lig induces structural defects within the material, concurrently compromising both stiffness and toughness.

**Fig. 7:**
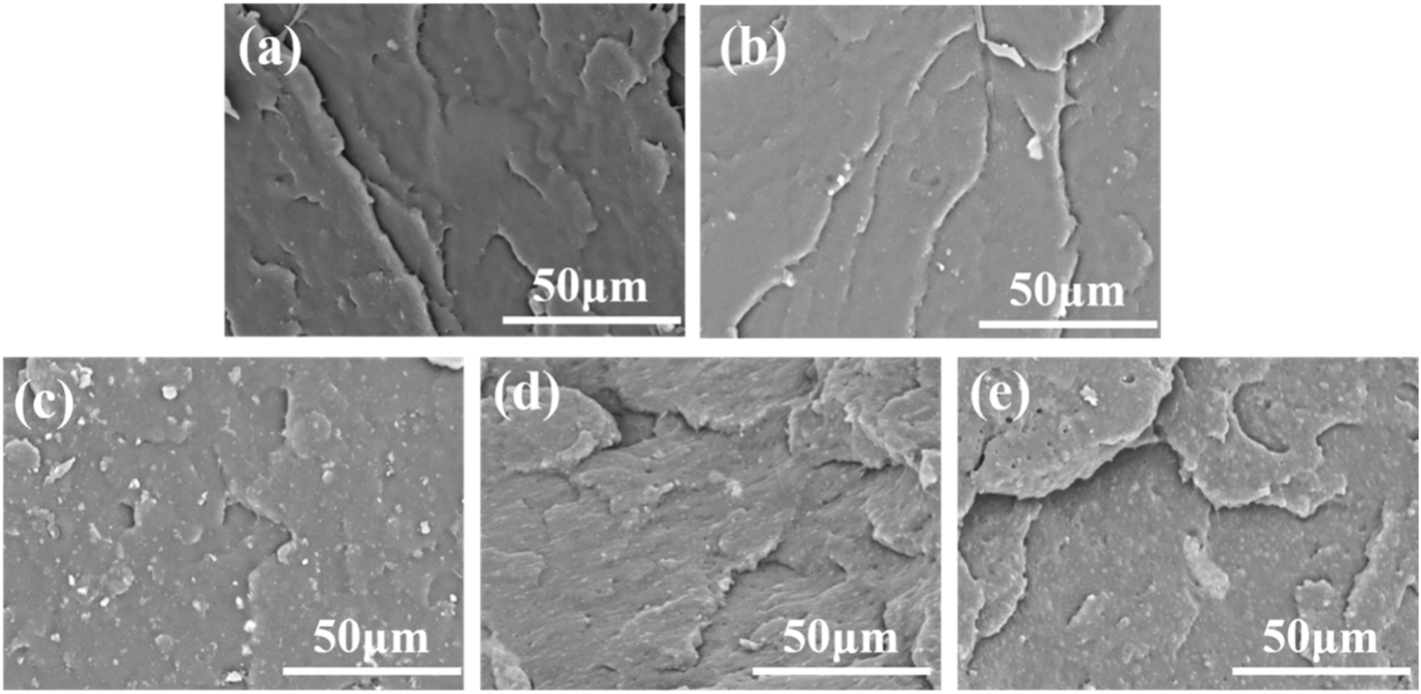
SEM images of the fracture surfaces after impact testing of T-PLA/P_2_N-lig: (a) T-PLA, (b) T-PLA/P_2_N-lig_3_, (c) T-PLA/P_2_N-lig_6_, (d) T-PLA/P_2_N-lig_9_, (e) T-PLA/P_2_N-lig_12_.

As evidenced by SEM images of impact-fractured surfaces (Fig. 7), increasing the P_2_N-lig content from 0 wt% to 12 wt% induces distinct morphological changes. Higher additive loadings result in the proliferation of fine particles and the formation of intricate crack networks, indicating progressive deterioration in P_2_N-lig dispersion homogeneity. Concurrently, elevated defect density and weakened interfacial compatibility amplify stress concentration effects at phase boundaries. These microstructural degradations collectively compromise the load-bearing capacity of the composite system, ultimately driving the observed reduction in mechanical performance.

The incorporation of P_2_N-lig into T-PLA composites significantly compromises their mechanical performance. While P_2_N-lig effectively enhances thermal stability and flame retardancy, its poor interfacial compatibility with the T-PLA matrix, aggravated interfacial stress concentration, and restricted molecular chain mobility collectively result in a progressive decline in mechanical properties with increasing additive content. At a 9 wt% P_2_N-lig loading, the composite achieves an optimal balance between flame retardancy and mechanical integrity, establishing this formulation as the functionally optimal candidate for practical applications requiring.

### 3.7 Mechanical and flame retardant properties analysis of T-PLA/P_2_N-lig FR 3D printing composites

**Fig. 8:**
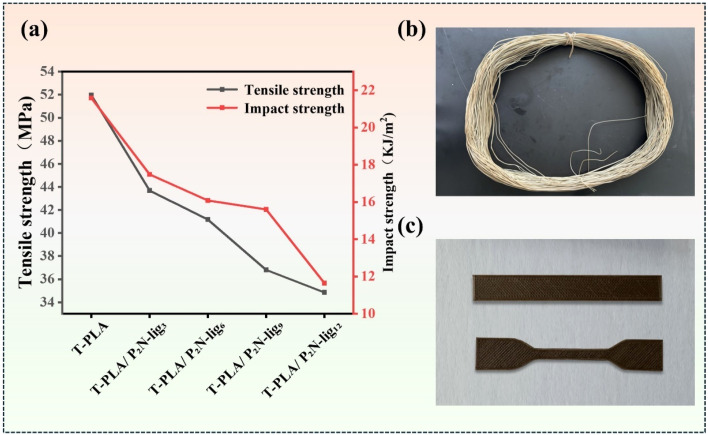
Mechanical properties of 3D-printed specimens: (a) mechanical property diagram, (b) 3D printing precursor, (c) 3D printed spline optical diagram.

The T-PLA/P2N-lig system was applied to 3D printing, and mechanical testing was conducted on the printed specimens. As illustrated in Fig. 8, the tensile strength and toughness (characterized by impact strength) of 3D-printed T-PLA and T-PLA/P_2_N-lig composites with varying P_2_N-lig loadings are presented. A comprehensive analysis of the observed trends and underlying mechanisms is provided below:

Pristine T-PLA exhibits a tensile strength of approximately 52 MPa, indicating robust mechanical properties. However, the tensile strength progressively declines with increasing P_2_N-lig content, reaching ∼34 MPa for the T-PLA/P_2_N-lig_12_ formulation. This deterioration is primarily attributed to the disruption of the ordered molecular chain arrangement in T-PLA caused by P_2_N-lig incorporation. During 3D printing, the inherent regularity of T-PLA chains contributes to its tensile performance, but P_2_N-lig additives compromise this structural order through interfacial incompatibility and molecular interaction effects. Poor compatibility between P_2_N-lig and the T-PLA matrix results in heterogeneous dispersion of additive particles at higher loadings, generating localized stress concentration points that initiate premature failure under tensile loading. Concurrently, interactions (*e.g.*, hydrogen bonding, steric hindrance) between P_2_N-lig and T-PLA chains alter interchain forces, restricting coordinated orientation and deformation during stretching, thereby diminishing load transfer efficiency and overall strength.

Pristine T-PLA demonstrates an impact strength of approximately 22 kJ m^−2^, indicating favorable toughness. The toughness decreases markedly with increasing P_2_N-lig content, with the T-PLA/P_2_N-lig_12_ formulation exhibiting a reduced impact strength of ∼12 kJ m^−2^. This degradation predominantly stems from interfacial effects introduced by P_2_N-lig. The toughness of 3D-printed specimens depends critically on structural homogeneity and resistance to crack propagation within the material. Incorporation of P_2_N-lig increases interfacial density within the composite, creating preferential pathways for crack initiation and extension under impact loading. At lower additive concentrations, interfacial effects remain negligible, but higher P_2_N-lig loadings amplify interfacial defects (*e.g.*, microvoids, particle agglomerates), facilitating rapid crack propagation and diminishing energy absorption capacity. Additionally, the inherent rigidity of P_2_N-lig particles and insufficient interfacial adhesion with the T-PLA matrix may induce interfacial debonding during impact events, further compromising the material's ability to dissipate energy.

Table 2 presents the flame-retardant properties of 3D-printed specimens, including limiting oxygen index (LOI) and UL-94 test results. Commercial PLA filaments (white, yellow, transparent) and pristine T-PLA exhibit LOI values ranging from 20.3% to 20.4%, indicating high flammability in ambient air and negligible flame retardancy. The incorporation of P_2_N-lig significantly enhances LOI values, increasing from 23.1% (T-PLA/P_2_N-lig_3_) to 25.9% (T-PLA/P_2_N-lig_12_), demonstrating a progressive improvement in flame retardancy with higher additive loadings, as evidenced by the elevated oxygen concentration required for combustion. In UL-94 testing, commercial PLA and T-PLA failed to achieve measurable ratings (*t*_1_, *t*_2_) due to poor flame retardancy, while P_2_N-lig-modified composites exhibited markedly reduced combustion times. For example, T-PLA/P_2_N-lig_6_ showed *t*_1_ = 19.7 s and *t*_2_ = 10.29 s, which further decreased to *t*_1_ = 9.65 s and *t*_2_ = 2.38 s for T-PLA/P_2_N-lig_12_, confirming P_2_N-lig's efficacy in suppressing combustion and promoting rapid self-extinguishment. Additionally, T-PLA/P_2_N-lig_6_, T-PLA/P_2_N-lig_9_, and T-PLA/P_2_N-lig_12_ all achieved a UL-94 V-2 rating, further validating the enhanced flame retardancy. Overall, P_2_N-lig incorporation markedly improves the flame retardancy of composites, with T-PLA/P_2_N-lig_6_ identified as the optimal formulation for 3D printing applications, balancing flame retardancy (LOI >24%, UL-94 V-2 rating) and mechanical performance retention (less pronounced mechanical property reduction).

**Table 2:** Flame retardancy of 3D-printed specimens

Samples	LOI (%)	*t*_1_ (s)	*t*_2_ (s)	Whether there are molten droplets	Rating
Commercial PLA filament (white)	20.3	—	—	Yes	—
Commercial PLA silk (yellow)	20.3	—	—	Yes	—
Commercial PLA silk (transparent)	20.4	—	—	Yes	—
T-PLA	20.4	—	—	Yes	—
T-PLA/P_2_N-lig_3_	23.1	—	—	Yes	—
T-PLA/P_2_N-lig_6_	24.1	19.7	10.29	Yes	V-2
T-PLA/P_2_N-lig_9_	25.2	17.27	7.66	Yes	V-2
T-PLA/P_2_N-lig_12_	25.9	9.65	2.38	Yes	V-2

## 4. Conclusions

In this work a green, high-efficiency, and low-cost lignin-based flame retardant was developed and was incorporated into modify toughened PLA for the fabrication of flame-retardant 3D printing materials. This composite demonstrates remarkable flame-retardant efficacy, achieving significant improvements in thermal stability and fire resistance at a 6 wt% additive loading while maintaining satisfactory mechanical performance. The optimized formulation (6 wt% flame retardant) exhibits a well-balanced profile of flame retardancy (LOI >24%, UL-94 V-2 rating) and mechanical integrity (tensile strength retention >85%, impact strength retention >70%), making it suitable for 3D printing applications. This strategy not only provides a novel approach for developing flame-retardant PLA composites but also significantly expands the utilization potential of lignin in advanced material systems, aligning with circular economy principles by valorizing biomass-derived resources.

## Author contributions

Siqi Ren: investigation, methodology, writing – review & editing, writing – original draft. Dongfang Fan: investigation, validation, data curation. Qian Zhang: investigation, validation, data curation. Yunhong Jiao: investigation, validation. Jianzhong Xu: conceptualization, resources. Jixing Xie: conceptualization, writing – review & editing, resources.

## Conflicts of interest

The authors declare that they have no known competing financial interests or personal relationships that could have appeared to influence the work reported in this paper.

## Supplementary Material

RA-015-D5RA06156C-s001

## Data Availability

The original contributions presented in the study are included in the article, further inquiries can be directed to the corresponding author. Supplementary information: elemental content table of flame retardants, the thermogravimetric table of flame retardants, the thermogravimetric table of splines with flame retardants added, and the conical data table of splines with flame retardants added. See DOI: https://doi.org/10.1039/d5ra06156c.
