# Supplementary material for: Fabrication of flame retardant toughened PLA base 3D printed materials with modified lignin by phytic acid
Source: RSC Adv. 2025 Oct 14;15(45):38294–306. doi: 10.1039/d5ra06156c (PMC12519074; doi:10.1039/d5ra06156c)
Supplement: RA-015-D5RA06156C-s001 [file RA-015-D5RA06156C-s001.pdf]

Supplementary Information (SI) for RSC Advances.
This journal is © The Royal Society of Chemistry 2025

Table S1. Element Content for P_2N-lig

Element	Weight Percentage	Atomic Percentage
C	28.61	36.60
O	50.74	48.74
N	7.33	8.04
P	13.33	6.61
Total Content	100	100

Table S2. Thermogravimetric data of P_2N-lig in N_2 atmosphere

Samples	$T_{5\%}$(℃)	T_{max}(℃)	Residual Mass（%）
Lignin	216.3	466.7	53.8
N-lig	228.3	294.5	11.6
P_2N-lig	232.7	287.8	37.1

Table S3. Thermogravimetric data of T-PLA/P_2N-lig in N_2 atmosphere

Samples	$T_{5\%}$(℃)	T_{max}(℃)	Residual Mass（%）
T-PLA	324.0	363.4	0.50
T-PLA/P_2N-lig_3	324.4	368.1	10.65
T-PLA/P_2N-lig_6	317.9	366.3	12.20
T-PLA/P_2N-lig_9	316.4	367.6	14.26
T-PLA/P_2N-lig_{12}	302.4	366.3	14.73

Table S4. Cone calorimetry data of T-PLA/P_2N-lig

Samples	T-PLA	T-PLA/P_2N-lig_3	T-PLA/P_2N-lig_6	T-PLA/P_2N-lig_9	T-PLA/P_2N-lig_{12}
TTI(s)	33	30	33	36	34
pHRR(kW/m^2)	691.04	529.74	490.33	419.63	371.84

THR(MJ/m^2)	86.60	74.34	72.79	66.79	65.08
Residue (%)	1.23	4.90	4.70	10.49	11.68
pSPR(m^2/s)	0.037	0.047	0.042	0.041	0.035
TSR(MJ/m^2)	350.12	608.05	603.47	614.67	525.13
COP(g/s)	0.0032	0.0034	0.0027	0.0039	0.0027
CO_2P(g/s)	0.579	0.398	0.405	0.351	0.300
